# Associations among Visual, Auditory, and Olfactory Functions in Community-Based Older Adults: The Atherosclerosis Risk in Communities (ARIC) Study

**DOI:** 10.1167/tvst.11.11.2

**Published:** 2022-11-02

**Authors:** Lubaina T. Arsiwala-Scheppach, Pradeep Y. Ramulu, A. Richey Sharrett, Vidyulata Kamath, Jennifer A. Deal, Xinxing Guo, Simo Du, Emmanuel E. Garcia Morales, Aleksandra Mihailovic, Honglei Chen, Alison G. Abraham

**Affiliations:** 1 Wilmer Eye Institute, Johns Hopkins School of Medicine, Johns Hopkins University, Baltimore, MD, USA; 2 Department of Epidemiology, Johns Hopkins Bloomberg School of Public Health, Johns Hopkins University, Baltimore, MD, USA; 3 Department of Psychiatry and Behavioral Sciences, Johns Hopkins School of Medicine, Johns Hopkins University, Baltimore, MD, USA; 4 Cochlear Center for Hearing and Public Health, Johns Hopkins Bloomberg School of Public Health, Johns Hopkins University, Baltimore, MD, USA; 5 Johns Hopkins Center on Aging and Health, Johns Hopkins Bloomberg School of Public Health, Johns Hopkins University, Baltimore, MD, USA; 6 Department of Epidemiology and Biostatistics, Michigan State University, East Lansing, MI, USA; 7 Department of Epidemiology, School of Public Health, University of Colorado, Denver, CO, USA; 8 Department of Ophthalmology, School of Medicine, University of Colorado, Denver, CO, USA

**Keywords:** visual acuity, contrast sensitivity, sensory impairment, race–sex interactions

## Abstract

**Purpose:**

Objective examination of relationships among visual, hearing, and olfactory function may yield mechanistic insights and inform our understanding of the burden of multiple-sensory impairments.

**Methods:**

This cross-sectional study capitalized on continuous measures of visual acuity (VA), contrast sensitivity, pure tone audiometry, Quick Speech-in-Noise (QuickSIN), and Sniffin’ Sticks from a subset of ARIC participants at two community sites (EyeDOC Study, 2017–2019). Scales of all measures were aligned such that higher values indicated greater impairment. Intersensory bivariate associations were assessed graphically, and correlations assessed using Kendall's tau. Intersensory associations, independent of age, education, smoking, diabetes, and hypertension, were examined using linear regression. Analyses were stratified by community/race (Washington County/White vs Jackson/Black) and sex (men vs women) to explore community–sex heterogeneity.

**Results:**

We included 834 participants (mean age, 79 years); 39% were from Jackson and 63% females. We found weak intersensory correlations (tau generally ≤0.15). In the demographics-adjusted regression models, results were heterogeneous across communities and sex. Worse near VA, contrast sensitivity, and olfaction were associated with worse QuickSIN and worse near VA was associated with worse olfaction in some but not all community/race–sex groups (e.g., Jackson/Black women, 0.1 logMAR worse near VA was associated with 0.27 units increase in QuickSIN [95% confidence interval, 0.10–0.45]). Associations were modestly attenuated by adjustment for the shared risk factors of smoking, diabetes, and hypertension.

**Conclusions:**

Visual dysfunction showed little or no association with hearing or olfaction impairments, suggesting a modest role for shared risk factors.

**Translational Relevance:**

Visually impaired individuals have only a modestly higher risk of other sensory impairment.

## Introduction

Visual impairment and age-related loss of other senses (i.e., hearing and olfaction) has a profound impact on how older adults live and function.^1^ Using standard thresholds for impairment, the prevalence of visual, hearing, and olfactory impairments individually has been reported to be 28%, 66%, and 50%, respectively, in adults aged 70 years or older.^2^^,^^3^ The projected burden of impairment in vision, hearing, and olfaction, and also dual visual and hearing impairments is expected to increase dramatically in the coming decades, consistent with population growth and longevity.^1^^,^^3^^–^^6^ Impairment in any single sensory domain is associated with a poorer quality of life and an increased risk of health hazards and dependency,^7^^–^^10^ but other senses can compensate for some loss. Multiple sensory impairment poses an even greater threat to one's daily functioning and quality of life.^2^ However, research on sensory deficits has largely focused on one or two senses at a time, because the loss of sensory function beyond a threshold for disability is rare compared with dysfunction of a single sense.^2^^,^^4^^,^^8^^–^^13^ Although multiple sensory impairment poses an even greater threat to one's daily functioning and quality of life, that is, by eliminating the ability to compensate for loss of sight via auditory substitution, it is an under-researched area.^2^

Multiple sensory impairment is increasingly common as individuals age.^1^ Beyond the strong association of all sensory impairments with age, chronic conditions such as diabetes increase the risk of both vision and hearing impairment,^11^^,^^14^ which suggests dual hearing and vision impairment may be more common than expected. However, research has also demonstrated cortical reorganization in the brain among single sensory-deprived individuals.^15^ Visual deprivation, for example, can lead to increased recruitment of the parietal cortex and superior colliculus by the auditory and somatosensory systems.^13^^,^^15^^,^^16^ Hence, sensory compensation for impairment in a single sense may actually improve performance in other senses, resulting in lower than expected dual sensory impairment. Elucidating relationships among visual, hearing, and olfactory impairments may, therefore, provide mechanistic insights regarding sensory loss, and provide clinicians information on the extent to which damage to one sense (i.e., visual impairment) increases the risk of dysfunction of other senses (i.e., hearing or olfaction), which can increase disability significantly.

Formal examinations of the relationships among vision, hearing, and olfaction have thus far been limited.^17^ Many studies lack objective measurements^12^ and studies vision are often limited to measurement of distance-visual acuity (VA),^12^ ignoring other aspects of visual function (near VA, contrast sensitivity [CS]). Studies have also neglected the potential impact of contextual or community factors such as access to health care, which may result in a greater burden of multisensory impairment as a result of untreated comorbidities or uncorrected refractive error.

To characterize the burden and co-occurrence of multiple sensory impairments across communities, we capitalized on objective measurements of visual, auditory, and olfactory functions collected from a bi-community sample of older adult participants in the Atherosclerosis Risk in Communities (ARIC) Study. We hypothesize that sensory function across the three sensory domains would be correlated positively, reflecting shared risk factors like smoking, diabetes, and hypertension, and that we would find heterogeneity across communities in sensory relationships. Elucidating relationships among visual, hearing, and olfactory impairments may provide mechanistic insights regarding sensory loss, informing sensory screening approaches, and help to identify subgroups with a higher burden of multiple sensory loss to better target health care.^6^

## Methods

### Study Population

The ARIC Study began as a prospective cohort of 15,792 participants, aged 45 to 64 years during the initial visit (1987–1989), with participants selected by probability sampling from four U.S. communities.^18^ We used visual measurements from the Eye Determinants of Cognition (EyeDOC) Study (2017–2019) (an ancillary study of the ARIC Study), hearing (audiometric and a measure of central auditory processing), and olfactory assessments from visit 6 (2016–2017) of the ARIC Study (Supplementary Fig. S1). The EyeDOC Study recruited current participants from two study sites: Jackson, Mississippi, where only Black participants were recruited, and Washington County, Maryland, which predominantly had White participants.^19^ The ARIC Study and ancillary studies that assess visual and other sensory functions adhered to the Declaration of Helsinki and were reviewed and approved by the institutional review boards of their respective study sites and the coordinating center. Written informed consent for the relevant studies was obtained from all participants.

### Assessment of Vision

Vision function was assessed at a single EyeDOC Study visit from May 17, 2017, to May 31, 2019.^20^ Most evaluations occurred between ARIC Study visits 6 and 7. Analyses were conducted using measurements from the better-performing eye because its function is more closely related to a person's daily functioning.^7^^,^^21^

#### Distance VA

Distance VA was assessed separately in the right and left eyes. Presenting distance VA was measured at four meters using a backlit Early Treatment Diabetic Retinopathy Study (ETDRS) chart and participants were instructed to wear their usual corrective lenses, if any. Corrected distance VA was measured using an autorefractor (Nidek ARK 560A or Topcon KR 800S) with built-in acuity charts. The number of letters correctly read in ETDRS presenting distance VA testing, and the lowest line with most letters read accurately for corrected distance VA testing, were then converted to the log_10_[minimum angle of resolution] (logMAR) scale. Better-eye presenting and corrected distance VA were derived as the respective lower (better) logMAR values between both eyes. For distance VA, 0.1 logMAR (the unit increment used for analyses) represented five letters or one line on the ETDRS chart and higher logMAR values indicate greater impairment.

#### Near VA

Near VA was assessed binocularly using sentences from the MNRead chart following standard procedures. Participants were instructed to read the sentences aloud as quickly and accurately as possible, with the MNRead chart placed at a viewing distance of 40 cm. Reading aids of +1.00 diopters (D) to +3.00 D in 0.50-D increments were provided, if needed. The number of sentences read and errors made for each sentence read were used to calculate the near VA for the participant on the logMAR scale. For near VA, 0.1 logMAR (the unit increment used for analyses) represented 1 line on the MNRead chart.

#### CS

CS was assessed separately in the right and left eye. It was tested using the Mars chart held at a distance of 50 cm with participants wearing their usual vision correction, if any.^22^^,^^23^ The number of letters correctly read were used to calculate CS for each eye on the logarithmic scale. Better-eye CS was derived as the higher (better) logarithmic value between both eyes.

### Assessment of Hearing

#### Peripheral Hearing

Pure tone audiometry (PTA) was conducted at ARIC Study visit 6 (2016–2017) in a sound-treated booth using insert earphones and an interacoustics AD629 audiometer (for on-site visits) or using a portable audiometer and supra-aural headphones (for home or long-term care facility visits).^24^ Air conduction thresholds in each ear were obtained at standard octaves from 0.5 kHz to 8 kHz by trained technicians. All thresholds were measured in decibels of hearing level. The PTA was calculated as the average of values of four frequencies (0.5 Hz, 1 Hz, 2 Hz, 4 Hz) in the better-hearing ear and higher values indicate greater impairment. The unit for PTA score is 1 dB. Analyses were conducted using measurements from the better-performing ear because its function may be more closely related to a person's daily functioning.

#### Central Auditory Processing

The Quick Speech-in-Noise (QuickSIN) test is composed of sentences recorded in four-talker babble and used to quantify a participant's ability to hear in background noise. Two sentence lists (track 14 and track 17) were provided. Each track is comprised of six sentences with five key words for each sentence. The background noise level for the first sentence was at 25 dB quieter than the speech and was increased by 5 dB with each subsequent sentence. The number of words correctly recognized was recorded for each sentence ranging from 0 to 5, for a possible range of 0 to 30 for each list. Performance on the two lists was averaged for analysis, where lower values indicate greater impairment.

### Assessment of Olfaction

Olfaction was measured via the 12-item Sniffin’ Sticks test at ARIC Study visit 6 (2016–2017).^25^ Participants were asked to smell 12 common odorants in odor-embedded felt-tip pens (orange, leather, cinnamon, peppermint, banana, lemon, licorice, coffee, cloves, pineapple, rose, and fish), one at a time, and asked to identify each pen using a multiple-choice format of four possible answer choices. One point was assigned to each correctly identified odorant, yielding a total possible score of 12. Hence, lower values indicate greater impairment.

### Covariates

Self-reported covariate data from ARIC Study visit 6 (2016–2017) were used, unless specified otherwise. Education level was assessed at visit 1 (1987–1989) and categorized as basic (less than high school), intermediate (high school graduate or vocational school), or advanced (at least some college, graduate school, or professional school). Smoking status was assessed as ever smoked cigarettes (yes or no).^26^^,^^27^ Diabetes was defined as measured fasting blood glucose levels of 126 mg/dL or greater, a nonfasting blood glucose level of 200 mg/dL or higher, taking hypoglycemic medication, or self-report of physician-diagnosed diabetes.^26^^–^^28^ Hypertension was defined as measured systolic blood pressure of 140 mm Hg or greater, a diastolic blood pressure of 90 mm Hg or greater, or taking antihypertensive medication.^26^^,^^27^

### Statistical Analyses

To facilitate the interpretation of our correlation and regression estimates, we aligned the direction of the scales of all measures such that higher (more positive) values indicated greater impairment across all scales, and a positive correlation always indicated better performance in one sense corresponded to better performance in the other sense. Hence, the original scales of three measures (CS, QuickSIN, and Sniffin’ Sticks) were reversed by multiplying by −1.

Participant characteristics at ARIC Study visit 6 (or the EyeDOC visit) were summarized for the entire cohort, by community/race and sex strata, and for excluded participants. Analyses were stratified by community/race and sex. We constructed scatterplots with locally weighted scatterplot smoothing to reveal trends in relationships between sensory functions. Kendall's tau, a nonparametric correlation metric, was used to evaluate the strength of the correlations.

Cross-sectional associations were also modeled using linear regression to allow for the effect of shared risk factors to be evaluated by comparing univariable and multivariable models. We constructed three nested models to understand the potential contribution of shared risk factors in the associations. Model 1 was adjusted for age only. Model 2 was additionally adjusted for education level (representing socioeconomic status). Model 3 was additionally adjusted for ever-smoker status (yes or no) and clinical conditions, namely, diabetes and hypertension (representing behavioral and morbidity factors).

Heterogeneity in relationships across communities was tested using models with community/race and sex three-way interaction terms (i.e., modification of the association of one sensory measure [i.e., exposure] with another sensory measure [i.e., outcome] by levels of community/race and sex). These models further built on model 3 by the addition of the following terms, interaction term of exposure with sex, interaction term of exposure with community/race, interaction term of community/race with sex, and interaction term of exposure with community/race with sex.

#### Sensitivity Analysis

To account for missing data, we repeated our regression analysis after performing multiple imputation by chained equations. Three imputed datasets were generated via the imputation strategy of predictive mean matching, using five iteration cycles for each dataset to impute the missing values. Linear regression analysis, as described elsewhere in this article, was performed on each imputed dataset. Finally, these regression results were pooled to obtain one regression estimate and the 95% confidence interval.

The level of significance was set to a *P* value of less than 0.05. All statistical analyses and data management were performed using RStudio (R version 4.0.2, The R Foundation for Statistical Computing, Vienna, Austria).

## Results

There were 834 participants who remained for analysis after excluding non-White participants from Washington County (*n* = 17) and those with missing sensory data (vision, *n* = 45; hearing, *n* = 73; olfaction, *n* = 41), demographic measures (age, sex, race, and education, *n* = 1), smoking status, or clinical comorbidities (*n* = 62). In our cohort, the mean age at ARIC Study visit 6 was 79 ± 4 years, 39% of participants were from the Jackson community (where all were Black), and 63% of the participants were women (Table 1). Compared with the 834 included participants, the 239 excluded participants were more likely to be Jackson/Black participants; had worse presenting distance VA, near VA, and olfaction; and had better PTA (Supplementary Table S1). The median time difference between the vision assessment (EyeDOC Study visit) and the hearing and olfaction assessments (ARIC Study visit 6) was 1.3 years (interquartile range, 1.1–1.6). Compared with participants in the other three community/race and sex strata, Jackson/Black men were more likely to have advanced education, diabetes, ever smoking, and worse near VA, whereas Jackson/Black women were more likely to have hypertension, but better presenting and corrected distance VA, near VA, PTA, and QuickSIN performances.

**Table 1.** Baseline Characteristics of the Entire Study Cohort Stratified by Community/Race and Sex

Characteristics	Jackson/Black Women	Washington County/White Women	Jackson/Black Men	Washington County/White Men	All
*N*	235	286	88	225	834
Age, years	78 ± 4	79 ± 4	77 ± 4	79 ± 4	79 ± 4
Education level					
Basic education	41 (17)	48 (17)	13 (15)	33 (15)	135 (16)
Intermediate education	73 (31)	144 (50)	18 (21)	109 (48)	344 (41)
Advanced education	121 (52)	94 (33)	57 (65)	83 (37)	355 (43)
Diabetes	98 (42)	103 (36)	39 (44)	80 (36)	320 (38)
Hypertension	215 (92)	220 (77)	73 (83)	175 (78)	683 (82)
Ever smoker	117 (50)	127 (44)	66 (75)	148 (66)	458 (55)
Presenting distance VA, logMAR^a^	0.14 ± 0.14	0.18 ± 0.14	0.17 ± 0.15	0.15 ± 0.17	0.16 ± 0.15
Corrected distance VA, logMAR^a^	0.04 ± 0.10	0.09 ± 0.12	0.05 ± 0.12	0.07 ± 0.14	0.07 ± 0.12
Near VA, logMAR^a^	0.14 ± 0.29	0.22 ± 0.17	0.32 ± 0.52	0.27 ± 0.22	0.22 ± 0.28
CS, log^b^	1.44 ± 0.15	1.40 ± 0.18	1.45 ± 0.20	1.43 ± 0.16	1.43 ± 0.17
PTA^a^	25 ± 10	31 ± 12	28 ± 14	39 ± 15	31 ± 14
QuickSIN^b^	20 ± 4	18 ± 5	18 ± 6	15 ± 6	18 ± 6
Sniffin’ Sticks^b^	8 ± 2	10 ± 2	8 ± 2	9 ± 2	9 ± 2

log, logarithm.

Note. In this table, all vision measures are in the scale of 1 unit. Values are mean ± standard deviation or number (%).

a Greater impairment is represented by higher values.

b Greater impairment is represented by lower values.

### Correlations

**Figure 1.**
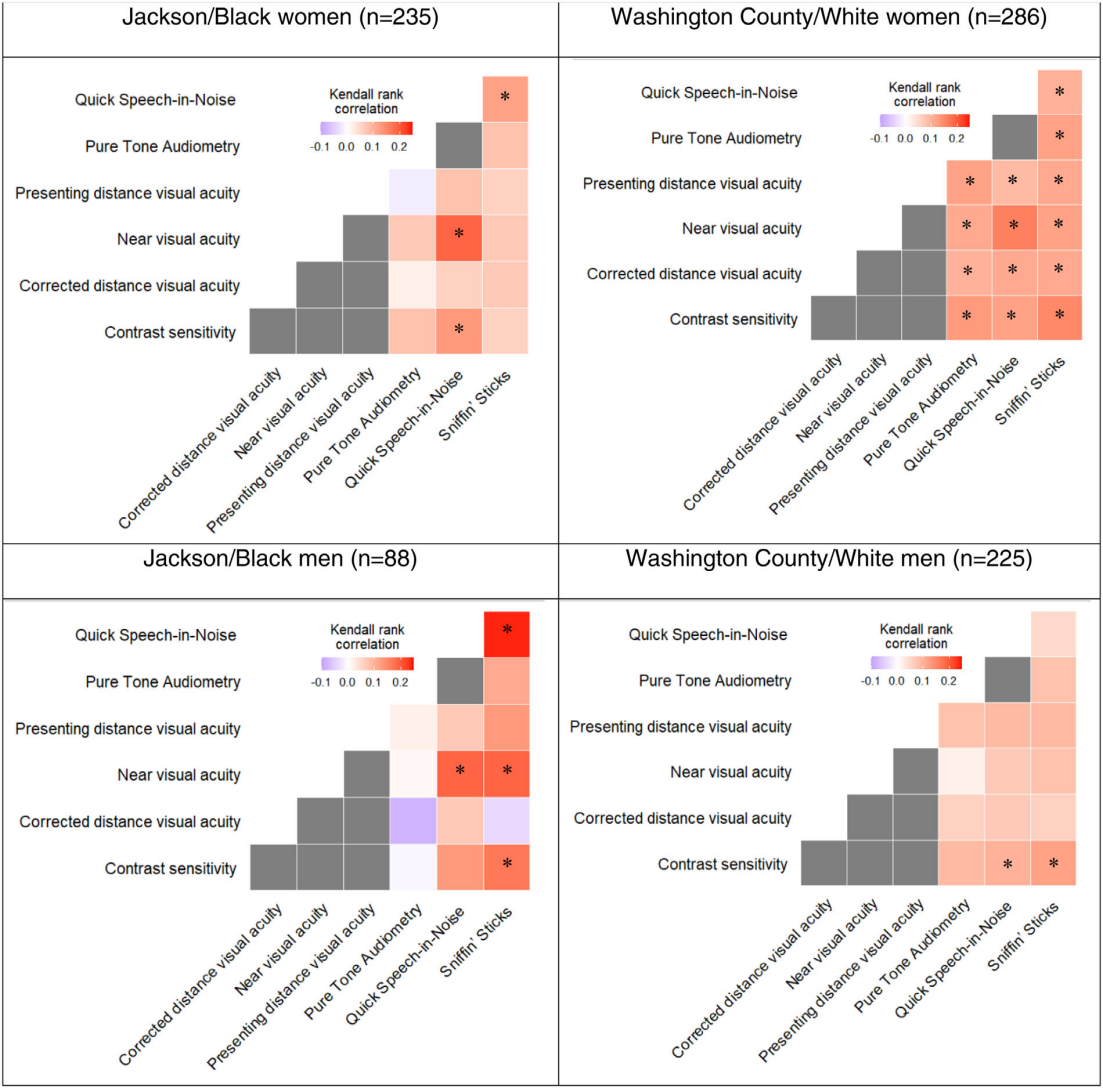
Pairwise Kendall rank correlation estimates between the different sensory functions, stratified by community/race and sex. Note. In these figures, for presenting and corrected distance VA, near VA, and PTA, worse function is represented by higher values. And for CS, QuickSIN, and Sniffin’ Sticks, the scale has been reversed such that greater impairment is represented by higher values. The gray cells represent intrasensory correlations and they were not examined in our study. *P < 0.05.

Correlations between the sensory modalities were generally weak in each community/race and sex stratum; with 89% of the tau absolute values being 0.15 or less (Fig. 1). However, for each pair of functions that was assessed, we generally observed positive correlations; for example, we found that worse visual function was correlated with worse auditory function. Compared with other visual measures, near VA showed the strongest correlation with QuickSIN in Washington County/White women (tau = 0.16; *P* < .001) and Jackson/Black participants (tau = 0.19 for men [*P* = 0.01] and tau = 0.19 for women [*P* < 0.001]). Furthermore, near VA and CS were both more strongly correlated with Sniffin’ Sticks in Jackson/Black men (tau = 0.19 [*P* = 0.02] and tau = 0.17 [*P* = 0.04], respectively) compared with other community/race and sex groups. Similarly, Sniffin’ Sticks was more strongly correlated with QuickSIN in Jackson/Black men (tau = 0.24; *P* = 0.002) compared with other community/race and sex groups. However, correlations remained weak across all community/race and sex subgroups.

### Regression Estimates

Table 2 and Supplementary Table S2 show regression estimates separately by community/race and sex. Poor vision showed scattered associations with poor hearing measured by QuickSIN and poor olfaction, independent of age (Supplementary Figs. S2–S5). Worse near VA and CS were associated with worse QuickSIN in three and two of the four community/race and sex groups, respectively. Worse presenting distance VA and near VA were associated with worse olfaction in one and three community/race and sex groups, respectively. There were no associations of vision with hearing measured by audiometry. Poor olfaction was associated with poor QuickSIN in two community/race and sex groups, independent of age. No significant associations were noted between hearing measured by audiometry and olfaction. These associations remained significant after the additional adjustment for education (model 2) in their respective community/race and sex groups.

**Table 2.** Linear Regression Estimates (95% Confidence Interval) of the Associations of Different Sensory Functions, Stratified by Community/Race and Sex, Which Are Statistically Significant in at Least One Community/Race or Sex Stratum

	Community/ Race	Jackson/Black Women	Washington County/White Women	Jackson/Black Men	Washington County/White Men
*N*		235	286	88	225
Exposure	Outcome	QuickSIN ^b^			
Near VA (0.1 logMAR worse)^a^	Model 1	0.36 (0.18 to 0.54)^d^	0.48 (0.16 to 0.80)^d^	0.32 (0.10 to 0.55)^d^	0.28 (−0.08 to 0.64)
	Model 2	0.27 (0.10 to 0.45)^d^	0.34 (0.03 to 0.66)^d^	0.26 (0.01 to 0.52)^d^	0.05 (−0.30 to 0.40)
	Model 3	0.28 (0.10 to 0.45)^d^	0.34 (0.03 to 0.66)^d^	0.27 (0.01 to 0.52)^d^	0.04 (−0.31 to 0.39)
CS (0.1 log worse)^b^	Model 1	0.43 (0.05 to 0.81)^d^	0.44 (0.13 to 0.74)^d^	0.25 (−0.38 to 0.88)	0.29 (−0.21 to 0.78)
	Model 2	0.40 (0.04 to 0.75)^d^	0.36 (0.07 to 0.66)^d^	−0.01 (−0.67 to 0.65)	0.17 (−0.29 to 0.64)
	Model 3	0.40 (0.04 to 0.76)^d^	0.37 (0.07 to 0.67)^d^	0.01 (−0.68 to 0.71)	0.17 (−0.29 to 0.64)
	Outcome	Sniffin’ Sticks^b^			
Presenting distance VA (0.1 logMAR worse)^a^	Model 1	0.12 (−0.10 to 0.36)	0.17 (−0.01 to 0.35)	0.25 (−0.06 to 0.56)	0.22 (0.03 to 0.41)^d^
	Model 2	0.12 (−0.11 to 0.35)	0.17 (−0.01 to 0.35)	0.16 (−0.17 to 0.49)	0.23 (0.04 to 0.42)^d^
	Model 3	0.11 (−0.12 to 0.34)	0.17 (−0.01 to 0.34)	0.08 (−0.27 to 0.42)	0.22 (0.03 to 0.41)^d^
Near VA (0.1 logMAR worse)^a^	Model 1	0.14 (0.03 to 0.24)^d^	0.13 (−0.02 to 0.28)	0.13 (0.04 to 0.22)^d^	0.17 (0.03 to 0.31)^d^
	Model 2	0.13 (0.03 to 0.24)^d^	0.13 (−0.02 to 0.28)	0.11 (0.01 to 0.21)^d^	0.21 (0.07 to 0.35)^d^
	Model 3	0.13 (0.02 to 0.24)^d^	0.12 (−0.03 to 0.26)	0.10 (−0.001 to 0.20)	0.21 (0.07 to 0.35)^d^
	Outcome	QuickSIN ^b^			
Sniffin’ Sticks (1 unit worse)^b^	Model 1	0.35 (0.13 to 0.57)^c^^,^^d^	0.09 (−0.17 to 0.34)^c^	0.73 (0.20 to 1.26)^c^^,^^d^	−0.07 (−0.42 to 0.27) ^c^
	Model 2	0.30 (0.09 to 0.51)^d^	0.09 (−0.16 to 0.33)	0.63 (0.09 to 1.16)^d^	0.03 (−0.29 to 0.35)
	Model 3	0.30 (0.09 to 0.51)^d^	0.11 (−0.14 to 0.36)	0.70 (0.10 to 1.21)^d^	to 0.05 (−0.28 to 0.37)

log, logarithm.

Note. All vision measures are in the scale of 0.1 unit. For presenting distance and near VA, 0.1 logMAR represented 5 letters or 1 line on the ETDRS chart, and 1 line on the MNRead chart, respectively. For CS, 0.1 log represented 2.5 letters on the MARS chart.

Model 1: adjusted for age.

Model 2: adjusted for age, education level.

Model 3: adjusted for age, education level, ever smoker status, diabetes, and hypertension.

a Greater impairment is represented by higher values.

b Scale has been reversed such that greater impairment is represented by higher values.

c Indicates significant exposure: community/race: sex interaction term in the relevant model, using the full cohort, additionally adjusted for sex, community/race, exposure : sex interaction, exposure: community/race interaction, community/race: sex interaction, and exposure: community/race: sex interaction.

d *P* < 0.05.

In all the pairwise associations between the different sensory functions, most estimates from models 2 and 3 were similar (i.e., additional adjustment of ever-smoker status, diabetes, and hypertension did not substantially attenuate the estimates). Where model 2 associations were statistically significant, model 3 never changed their magnitude by more than 11%.

### Sensitivity Analysis

The regression results obtained from the imputed datasets (multiple imputation by chained equations analysis) were largely similar to those obtained from the original datasets, that is, the primary analysis (Supplementary Table S3).

## Discussion

In our study of community-based older adults aged 71 to 93 years, we observed that worse performance in visual measures were correlated with worse performance in central auditory processing and olfaction, but those correlations were weak. Although shared risk factors were expected to lead to positive correlations, and indeed correlations among the measures of vision, hearing, and olfactory function were generally positively correlated, the weak univariable relationships were not notably changed by adjustment for potential shared risk factors for sensory impairment (i.e., age, education, smoking, diabetes, and hypertension). We had hypothesized that sensory impairments may be linked through factors like smoking status, diabetes, and hypertension, which can lead to vascular damage that could affect vision, hearing, and potentially olfaction. Our findings suggest that in older adults aged 71 to 93 years, smoking status, diabetes, and hypertension are not strong risk factors for shared sensory impairments and that loss of vision, hearing, and olfaction occur most often as a result of different pathogenic processes. The weak correlations and associations between different sensory functions in our cohort suggest that damage to multiple sensory functions occurs largely independent of damage within the individual senses. Furthermore, the participants from Washington County (White) and Jackson (Black) sites of the ARIC Study recruited in our study differed by racial and community-based factors. These two communities differed by geographic location, urbanicity, socioeconomic characteristics, Area Deprivation Index (ADI), and Air Quality Index (by the U.S. Environmental Protection Agency). The ADI are created for entire United States by the Health Resources & Services Administration and the University of Wisconsin School of Medicine and Public Health. The ADI are national percentile rankings at the Census Block Group or neighborhood level from 1 (i.e., lowest level of disadvantage within the nation) to 100 (i.e., highest level of disadvantage within the nation). Hagerstown, Washington County, Maryland, is a largely rural, more northern area with an average ADI of 74 and with 29 unhealthy days in 2011 through 2020 regarding pollutants that affect older adults; that is, the Air Quality Index exceeded values of 100, whereas Jackson, Mississippi, is a city in the southern United States with an average ADI of 100 and with 13 unhealthy days in 2011 through 2020 regarding pollutants that affect older adults; that is, the Air Quality Index exceeded values of 100.^29^^–^^33^ These factors may also influence the kind of infrastructure, services, and opportunities available to older adults for outdoor physical activities, educational and social interactions, and so on, which are important for maintaining health holistically.

Two recent studies^34^^,^^35^ found that vision, hearing, and olfactory impairments were associated with poor cognition independent of each other, highlighting the potential involvement of the brain. In our results, the finding of statistical significance in some associations using the QuickSIN measure of central auditory processing, but not in the audiometric hearing measure, may suggest that the correlation stemmed from involvement of the brain's sensory association areas. Also, the Sniffin’ Sticks test used in our study is an odor identification task that demands a higher cognitive load than, for example, odor detection threshold tasks.^36^^,^^37^

A potential mechanism linking sensory function impairments is through shared risk factors. For example, diabetes, hypertension, and smoking have been associated with several individual eye diseases and worse vision in population-based studies.^14^^,^^38^^–^^41^ In all the pairwise associations between the different sensory functions (i.e., vision vs hearing, vision vs olfaction, and hearing vs olfaction), we observed that the additional adjustment of ever-smoker status, diabetes, and hypertension had only a trivial effect on the regression estimates, in comparison with the simpler age- and education-adjusted model. This finding may suggest that in older adults aged 71 to 93 years, smoking status, diabetes, and hypertension are not strong risk factors for shared sensory impairments and that, in this cohort for this age group, decreases in these sensory functions may result from different undefined pathogenic processes.

The prevalence of sensory impairments is an important consideration when quantifying the associations between the different sensory functions. Using thresholds to quantify the prevalence of sensory impairments, Armstrong et al.^42^ observed that vision, hearing, and olfactory loss were common (each >10%) in the ARIC Study cohort, which was evaluated in the prepriesent study. Our results show that visual impairment was more common in Jackson/Black participants. In contrast, in line with two previous studies,^4^^,^^38^ we observed that audiometric hearing loss was more prevalent in men and in Washington County/White participants. A hypothesis for the higher prevalence of audiometric hearing loss in White participants as compared with Black, as put forth in a previous study,^43^ is that skin pigmentation of the ear, a marker of melanocytic function, may be protective against hearing loss in Black participants. In accordance with another study,^6^ we observed that Jackson/Black participants had worse olfactory performance as compared with Washington County/Whites participants. In contrast with the results of a meta-analysis of sex differences in olfactory performance,^44^ we did not observe a female advantage for odor identification performance. It is noteworthy that, despite reports in literature,^1^^,^^2^ we did not find any meaningful or consistent differences among the four community/race and sex groups in these associations in our cohort. We would like to remind the readers that in this study, the community/race strata represent a conglomerate of contextual differences between the two communities that could drive differences in sensory loss and risk factors for systemic disease that may cause such loss.

The strengths of our study are, first, that results were derived from two racially distinct communities with a large cohort of older adults aged 71 to 93 years. Second, multiple measures of visual function were used to examine its different aspects. Third, all sensory assessments were conducted following standard protocols, hence decreasing imprecision in our measures. However, our results should be interpreted in light of the following limitations. Our cohort included White and Black races only, and race and community were inextricably connected, which prevented differentiation of the effects of race from geographic location. We cannot separate out the effect of structural racism from housing instability, for example. Hence, the study results should be generalized in the context of race as a social construct, capturing many of the same structural and contextual factors that are captured by neighborhood or community, rather than race being viewed as a biological factor. Second, we assessed a single aspect of olfactory functioning, that is, odor identification.

Our results have important implications. It seems that the loss of vision, hearing, and smell do not generally arise from the common lifestyle and comorbid factors investigated in our study population. This finding suggests that local factors, like structural damage, inflammation, and infection, more often lead to a decrease in vision, central auditory processing, and olfactory function. Even in the light of unshared risk factors for the different sensory impairments, the loss of function in one sense can likely impact the other senses in a more dramatic manner. Our results show that the prevalence of visual impairments with other sensory impairments differs by community/race and sex strata. Furthermore, any form of sensory impairment (single, dual, or global sensory impairment) has been tied to decreased physical, mental, and social well-being and increased risk of mortality.^2^^,^^45^^,^^46^ Affected individuals are likely to experience psychosocial isolation arising from communication breakdown and/or the inability to participate in social interactions.^47^ The resulting isolation may lead to depression, anxiety, lethargy, and social dissatisfaction.^48^

## Supplementary Material

Supplement 1

## References

[1] Correia C, Lopez KJ, Wroblewski KE, et al. Global sensory impairment in older adults in the United States. *J Am Geriatr Soc*. 2016; 64(2): 306–313, doi:10.1111/jgs.13955. 26889840 PMC4808743

[2] Brenowitz WD, Kaup AR, Lin FR, Yaffe K. Multiple sensory impairment is associated with increased risk of dementia among black and white older adults. *J Gerontol A Biol Sci Med Sci*. 2019; 74(6): 890–896, doi:10.1093/gerona/gly264. 30452551 PMC6521912

[3] Doty RL, Kamath V. The influences of age on olfaction: a review. *Front Psychol*. 2014; 5: 20, doi:10.3389/fpsyg.2014.00020. 24570664 PMC3916729

[4] Lin FR, Thorpe R, Gordon-Salant S, Ferrucci L. Hearing loss prevalence and risk factors among older adults in the United States. *J Gerontol A Biol Sci Med Sci*. 2011; 66(5): 582–590, doi:10.1093/gerona/glr002. 21357188 PMC3074958

[5] Bourne RRA, Flaxman SR, Braithwaite T, et al. Magnitude, temporal trends, and projections of the global prevalence of blindness and distance and near vision impairment: a systematic review and meta-analysis. *Lancet Glob Health*. 2017; 5(9): e888–e897, doi:S2214-109X(17)30293-0. 28779882 10.1016/S2214-109X(17)30293-0

[6] Pinto JM, Schumm LP, Wroblewski KE, Kern DW, McClintock MK. Racial disparities in olfactory loss among older adults in the United States. *J Gerontol A Biol Sci Med Sci*. 2014; 69(3): 323–329, doi:10.1093/gerona/glt063. 23689829 PMC3976135

[7] Broman AT, Munoz B, Rodriguez J, et al. The impact of visual impairment and eye disease on vision-related quality of life in a Mexican-American population: Proyecto VER. *Invest Ophthalmol Vis Sci*. 2002; 43(11): 3393–3398. 12407148

[8] Santos DV, Reiter ER, DiNardo LJ, Costanzo RM. Hazardous events associated with impaired olfactory function. *Arch Otolaryngol Head Neck Surg*. 2004; 130(3): 317–319, doi:10.1001/archotol.130.3.317. 15023839

[9] Aschenbrenner K, Hummel C, Teszmer K, et al. The influence of olfactory loss on dietary behaviors. *Laryngoscope*. 2008; 118(1): 135–144, doi:10.1097/MLG.0b013e318155a4b9. 17975508

[10] Deal JA, Reed NS, Kravetz AD, et al. Incident hearing loss and comorbidity: a longitudinal administrative claims study. *JAMA Otolaryngol Head Neck Surg*. 2019; 145(1): 36–43, doi:10.1001/jamaoto.2018.2876. 30419134 PMC6439817

[11] Kim MB, Zhang Y, Chang Y, et al. Diabetes mellitus and the incidence of hearing loss: A cohort study. *Int J Epidemiol*. 2017; 46(2): 717–726, doi:10.1093/ije/dyw243. 27818377 PMC6251644

[12] Heine C, Browning C. Dual sensory loss in older adults: a systematic review. *Gerontologist*. 2015; 55(5): 913–928, doi:10.1093/geront/gnv074. 26315316

[13] Sorokowska A, Sorokowski P, Karwowski M, Larsson M, Hummel T. Olfactory perception and blindness: a systematic review and meta-analysis. *Psychol Res*. 2019; 83(8): 1595–1611, doi:10.1007/s00426-018-1035-2. 29948185 PMC6794238

[14] Duh EJ, Sun JK, Stitt AW. Diabetic retinopathy: current understanding, mechanisms, and treatment strategies. *JCI Insight*. 2017; 2(14): eCollection 2017 Jul 20, doi:10.1172/jci.insight.93751. PMC5518557 28724805

[15] Merabet LB, Pascual-Leone A. Neural reorganization following sensory loss: the opportunity of change. *Nat Rev Neurosci*. 2010; 11(1): 44–52, doi:10.1038/nrn2758. 19935836 PMC3898172

[16] Neville H, Bavelier D. Human brain plasticity: evidence from sensory deprivation and altered language experience. *Prog Brain Res*. 2002; 138: 177–188, doi:S0079-6123(02)38078-6 [pii]. 12432770 10.1016/S0079-6123(02)38078-6

[17] Adams DR, Wroblewski KE, Kern DW, et al. Factors associated with inaccurate self-reporting of olfactory dysfunction in older US adults. *Chem Senses*. 2017; 42(3): 223–231, doi:10.1093/chemse/bjw108. 28007787 PMC6074942

[18] Wright JD, Folsom AR, Coresh J, et al. The ARIC (Atherosclerosis Risk in Communities) study: JACC focus seminar 3/8. *J Am Coll Cardiol*. 2021; 77(23): 2939–2959, doi:S0735-1097(21)04788-4 [pii]. 34112321 10.1016/j.jacc.2021.04.035 PMC8667593

[19] Abraham AG, Guo X, Arsiwala LT, et al. Cognitive decline in older adults: what can we learn from optical coherence tomography (OCT)-based retinal vascular imaging? *J Am Geriatr Soc*. 2021; 69(9): 2524–2535, doi:10.1111/jgs.17272. 34009667 PMC8440348

[20] Arsiwala LT, Guo X, Ramulu PY, et al. Associations of visual function with cognitive performance in community-based older adults: the Eye Determinants of Cognition study. *J Gerontol A Biol Sci Med Sci*. 2022; 77(10): 2133–2140, doi:glab349 [pii]. 35089306 10.1093/gerona/glab349 PMC9536449

[21] Rubin GS, Munoz B, Bandeen-Roche K, West SK. Monocular versus binocular visual acuity as measures of vision impairment and predictors of visual disability. *Invest Ophthalmol Vis Sci*. 2000; 41(11): 3327–3334. 11006221

[22] Arditi A. Improving the design of the letter contrast sensitivity test. *Invest Ophthalmol Vis Sci*. 2005; 46(6): 2225–2229. 10.1167/iovs.04-1198. Accessed 19 July, 2022. 15914645

[23] Mars Perceptrix Corporation. The Mars letter contrast sensitivity test. Available at: https://www.marsperceptrix.com/sites/default/files/downloads/MarsLetterCSTestUserManualEnglish.pdf. Updated 2013. Accessed July 19, 2022.

[24] Deal JA, Sharrett AR, Albert MS, et al. Hearing impairment and cognitive decline: a pilot study conducted within the Atherosclerosis Risk in Communities Neurocognitive study. *Am J Epidemiol*. 2015; 181(9): 680–690, doi:10.1093/aje/kwu333. 25841870 PMC4408947

[25] Palta P, Chen H, Deal JA, et al. Olfactory function and neurocognitive outcomes in old age: the Atherosclerosis Risk in Communities Neurocognitive study. *Alzheimers Dement*. 2018; 14(8): 1015–1021, doi:S1552-5260(18)30073-6 [pii]. 29605223 10.1016/j.jalz.2018.02.019 PMC6097922

[26] ARIC Study. ARIC home and field center procedures ARIC-NCS visit 6 study protocol. Available at: https://sites.cscc.unc.edu/aric/sites/default/files/public/manuals/MOP2%207.7%20%2B%20Appendices.pdf. Updated 2017. Accessed July 19, 2022.

[27] Liao D, Cai J, Rosamond WD, et al. Cardiac autonomic function and incident coronary heart disease: a population-based case-cohort study. the ARIC study. Atherosclerosis Risk in Communities study. *Am J Epidemiol*. 1997; 145(8): 696–706, doi:10.1093/aje/145.8.696. 9125996

[28] ARIC Study. ARIC Manual 30 ARIC analysis manual. Available at: https://sites.cscc.unc.edu/aric/sites/default/files/public/manuals/ARIC%20Manual%2030_200207.pdf. Updated 2020. Accessed July 19, 2022.

[29] Naorungroj S, Slade GD, Divaris K, Heiss G, Offenbacher S, Beck JD. Racial differences in periodontal disease and 10-year self-reported tooth loss among late middle-aged and older adults: the dental ARIC study. *J Public Health Dent*. 2017; 77(4): 372–382, doi:10.1111/jphd.12226. 28585323 PMC5718983

[30] Naorungroj S, Schoenbach VJ, Wruck L, et al. Tooth loss, periodontal disease, and cognitive decline in the Atherosclerosis Risk in Communities (ARIC) study. *Community Dent Oral Epidemiol*. 2015; 43(1): 47–57, doi:10.1111/cdoe.12128. 25363061 PMC4303516

[31] Borrell LN, Beck JD, Heiss G. Socioeconomic disadvantage and periodontal disease: the dental Atherosclerosis Risk in Communities study. *Am J Public Health*. 2006; 96(2): 332–339, doi:AJPH.2004.055277 [pii]. 16380570 10.2105/AJPH.2004.055277 PMC1470476

[32] Kind AJH, Buckingham W. Making neighborhood disadvantage metrics accessible: the Neighborhood Atlas. *N Engl J Med.* 2018; 378: 2456–2458, doi:10.1056/NEJMp1802313. PMCID: PMC6051533. AND University of Wisconsin School of Medicine and Public Health. 2015 Area Deprivation Index v2.0. Downloaded from https://www.neighborhoodatlas.medicine.wisc.edu/. Accessed Jul 19, 2022. 29949490 PMC6051533

[33] U.S. EPA Office of Air Quality Planning and Standards. AirNow - U.S. air quality index. Available at: https://gispub.epa.gov/airnow/?contours=ozonepm&tab=current&monitors=ozonepm&showlegend=yes&xmin=-19377321.493042495&xmax=-6100515.42802405&ymin=3951206.5422114665&ymax=13001350.69117393. Accessed July 19, 2022.

[34] Fischer ME, Cruickshanks KJ, Schubert CR, et al. Age-related sensory impairments and risk of cognitive impairment. *J Am Geriatr Soc*. 2016; 64(10): 1981–1987, doi:10.1111/jgs.14308. 27611845 PMC5073029

[35] Schubert CR, Cruickshanks KJ, Fischer ME, et al. Sensory impairments and cognitive function in middle-aged adults. *J Gerontol A Biol Sci Med Sci*. 2017; 72(8): 1087–1090, doi:10.1093/gerona/glx067. 28535277 PMC5861860

[36] Calhoun-Haney R, Murphy C. Apolipoprotein epsilon4 is associated with more rapid decline in odor identification than in odor threshold or dementia rating scale scores. *Brain Cogn*. 2005; 58(2): 178–182, doi:S0278-2626(04)00307-0 [pii]. 15919549 10.1016/j.bandc.2004.10.004

[37] Hedner M, Larsson M, Arnold N, Zucco GM, Hummel T. Cognitive factors in odor detection, odor discrimination, and odor identification tasks. *J Clin Exp Neuropsychol*. 2010; 32(10): 1062–1067, doi:10.1080/13803391003683070. 20437286

[38] Munoz B, West SK, Rubin GS, et al. Causes of blindness and visual impairment in a population of older Americans: the Salisbury Eye Evaluation study. *Arch Ophthalmol*. 2000; 118(6): 819–825, doi:eeb90018 [pii]. 10865321 10.1001/archopht.118.6.819

[39] Bhargava M, Ikram MK, Wong TY. How does hypertension affect your eyes? *J Hum Hypertens*. 2012; 26(2): 71–83, doi:10.1038/jhh.2011.37. 21509040

[40] Cheng AC, Pang CP, Leung AT, Chua JK, Fan DS, Lam DS. The association between cigarette smoking and ocular diseases. *Hong Kong Med J*. 2000; 6(2): 195–202. 10895144

[41] Zhang X, Kahende J, Fan AZ, et al. Smoking and visual impairment among older adults with age-related eye diseases. *Prev Chronic Dis*. 2011; 8(4): A84, doi:A84 [pii]. 21672408 PMC3136979

[42] Armstrong NM, Wang H, JY E, et al. Patterns of prevalence of multiple sensory impairments among community-dwelling older adults. *J Gerontol A Biol Sci Med Sci*. 2021; 77(10): 2123–2132, doi:glab294 [pii]. 10.1093/gerona/glab294 PMC9536434 34608938

[43] Lin FR, Maas P, Chien W, Carey JP, Ferrucci L, Thorpe R. Association of skin color, race/ethnicity, and hearing loss among adults in the USA. *J Assoc Res Otolaryngol*. 2012; 13(1): 109–117, doi:10.1007/s10162-011-0298-8. 22124888 PMC3254716

[44] Sorokowski P, Karwowski M, Misiak M, et al. Sex differences in human olfaction: a meta-analysis. *Front Psychol*. 2019; 10: 242, doi:10.3389/fpsyg.2019.00242. 30814965 PMC6381007

[45] Pinto JM, Wroblewski KE, Huisingh-Scheetz M, et al. Global sensory impairment predicts morbidity and mortality in older U.S. adults. *J Am Geriatr Soc*. 2017; 65(12): 2587–2595, doi:10.1111/jgs.15031. 28942611 PMC6317884

[46] Schubert CR, Fischer ME, Pinto AA, et al. Sensory impairments and risk of mortality in older adults. *J Gerontol A Biol Sci Med Sci*. 2017; 72(5): 710–715, doi:10.1093/gerona/glw036. 26946102 PMC5861964

[47] Schneider JM, Gopinath B, McMahon CM, Leeder SR, Mitchell P, Wang JJ. Dual sensory impairment in older age. *J Aging Health*. 2011; 23(8): 1309–1324, doi:10.1177/0898264311408418. 21596997

[48] Heine C, Browning CJ. Communication and psychosocial consequences of sensory loss in older adults: overview and rehabilitation directions. *Disabil Rehabil*. 2002; 24(15): 763–773, doi:10.1080/09638280210129162. 12437862

