# Supplementary material for: Associations among Visual, Auditory, and Olfactory Functions in Community-Based Older Adults: The Atherosclerosis Risk in Communities (ARIC) Study
Source: Transl Vis Sci Technol. 2022 Nov 2;11(11):2. doi: 10.1167/tvst.11.11.2 (PMC9639698; doi:10.1167/tvst.11.11.2)
Supplement: Supplement 1 [file tvst-11-11-2_s001.pdf]

SUPPLEMENTARY MATERIAL

Supplementary Table S1. Baseline characteristics of the excluded and included participants

Characteristics	Excluded participants	Included participants	P-value, test of difference
n	239	834	
Age, mean (SD), years	79 (5)	79 (4)	0.36
Women, n (%)	151 (66)	521 (63)	0.41
Jackson/Black participants, n (%)	176 (77)	323 (39)	<0.001
Education level, n (%)			0.30
Basic education	48 (20)	135 (16)	
Intermediate education	89 (37)	344 (41)	
Advanced education	101 (42)	355 (43)	
Diabetes, n (%)	91 (46)	320 (38)	0.05
Hypertension, n (%)	193 (87)	683 (82)	0.12
Ever-smoker, n (%)	110 (63)	458 (55)	0.05
Presenting distance visual acuity, mean (SD), logMAR *	0.19 (0.23)	0.16 (0.15)	0.005
Corrected distance visual acuity, mean (SD), logMAR *	0.09 (0.20)	0.07 (0.12)	0.05
Near visual acuity, mean (SD), logMAR *	0.34 (0.52)	0.22 (0.28)	<0.001
Contrast sensitivity, mean (SD), log †	1.41 (0.20)	1.43 (0.17)	0.17
Pure Tone Audiometry, mean (SD) *	29 (12)	31 (14)	0.04
Quick Speech-in-Noise, mean (SD) †	17 (6)	18 (6)	0.28
Sniffin' Sticks, mean (SD) †	8 (2)	9 (2)	0.001

Note. In this table, all vision measures are in the scale of 1 unit.
* Greater impairment is represented by higher values.
† Greater impairment is represented by lower values.
logMAR: $\log_{10}$[minimum angle of resolution]; log: logarithm

Supplementary Table S2. Linear regression estimates (95% confidence interval) of the associations of different sensory functions, stratified by community/race-sex, which are statistically significant in no community/race-sex strata, beyond the age-adjusted model (Model 1)

	Community/race	Jackson/Black women	Washington County/White women	Jackson/Black men	Washington County/White men
n		235	286	88	225
Exposure	Outcome	Pure Tone Audiometry *			
Presenting distance visual acuity (0.1 logMAR worse) *	Model 1	-0.09 (-1.03, 0.85)	0.59 (-0.41, 1.59)	-0.64 (-2.54, 1.27)	0.60 (-0.57, 1.77)
	Model 2	-0.11 (-1.06, 0.84)	0.60 (-0.41, 1.61)	-1.24 (-3.27, 0.79)	0.44 (-0.70, 1.58)
	Model 3	-0.13 (-1.09, 0.83)	0.62 (-0.40, 1.64)	-1.22 (-3.36, 0.92)	0.42 (-0.73, 1.58)
Corrected distance visual acuity (0.1 logMAR worse) *	Model 1	-0.45 (-1.81, 0.89)	0.28 (-0.90, 1.46)	-1.15 (-3.62, 1.32)	0.74 (-0.71, 2.18)
	Model 2	-0.49 (-1.84, 0.86)	0.28 (-0.90, 1.47)	-1.79 (-4.36, 0.78)	0.58 (-0.83, 1.98)
	Model 3	-0.51 (-1.87, 0.86)	0.35 (-0.85, 1.55)	-1.58 (-4.28, 1.13)	0.55 (-0.87, 1.96)
Near visual acuity (0.1 logMAR worse) *	Model 1	0.35 (-0.07, 0.78)	0.70 (-0.13, 1.52)	0.44 (-0.12, 1.00)	0.30 (-0.56, 1.16)
	Model 2	0.33 (-0.11, 0.78)	0.73 (-0.12, 1.57)	0.35 (-0.26, 0.97)	-0.11 (-0.97, 0.75)
	Model 3	0.33 (-0.11, 0.78)	0.70 (-0.15, 1.55)	0.38 (-0.25, 1.01)	-0.10 (-0.97, 0.76)
Contrast sensitivity (0.1 log worse) †	Model 1	0.44 (-0.45, 1.34)	0.63 (-0.16, 1.42)	-0.38 (-1.86, 1.10)	0.81 (-0.37, 1.99)
	Model 2	0.43 (-0.47, 1.33)	0.64 (-0.16, 1.43)	-0.82 (-2.40, 0.75)	0.62 (-0.54, 1.77)
	Model 3	0.44 (-0.48, 1.35)	0.68 (-0.13, 1.48)	-0.65 (-2.30, 1.00)	0.60 (-0.56, 1.76)
	Outcome	Quick Speech-in-Noise †			
Presenting distance visual acuity (0.1 logMAR worse) *	Model 1	0.41 (0.01, 0.81) §	0.34 (-0.05, 0.73)	0.13 (-0.68, 0.94)	0.29 (-0.20, 0.78)
	Model 2	0.35 (-0.03, 0.73)	0.24 (-0.14, 0.62)	-0.24 (-1.09, 0.61)	0.20 (-0.26, 0.66)
	Model 3	0.35 (-0.03, 0.73)	0.26 (-0.12, 0.64)	-0.28 (-1.18, 0.63)	0.19 (-0.27, 0.66)
Corrected distance	Model 1	0.44 (-0.13, 1.01)	0.33 (-0.13, 0.79)	0.40 (-0.65, 1.45)	0.26 (-0.35, 0.86)

visual acuity (0.1 logMAR worse) *	Model 2	0.36 (-0.18, 0.90)	0.24 (-0.20, 0.69)	0.04 (-1.04, 1.13)	0.16 (-0.40, 0.73)
	Model 3	0.32 (-0.22, 0.86)	0.25 (-0.20, 0.70)	0.06 (-1.09, 1.21)	0.16 (-0.41, 0.73)
	Outcome	Sniffin' Sticks †			
Corrected distance visual acuity (0.1 logMAR worse) *	Model 1	0.23 (-0.09, 0.57)	0.14 (-0.07, 0.35)	-0.003 (-0.41, 0.41)	0.14 (-0.09, 0.37)
	Model 2	0.23 (-0.10, 0.56)	0.14 (-0.07, 0.34)	-0.14 (-0.56, 0.28)	0.15 (-0.08, 0.38)
	Model 3	0.21 (-0.12, 0.54)	0.17 (-0.04, 0.38)	-0.27 (-0.70, 0.16)	0.14 (-0.09, 0.37)
Contrast sensitivity (0.1 log worse) †	Model 1	-0.03 (-0.24, 0.19)	0.10 (-0.03, 0.24)	0.11 (-0.14, 0.35)	0.13 (-0.06, 0.32)
	Model 2	-0.03 (-0.25, 0.19)	0.10 (-0.04, 0.24)	0.02 (-0.24, 0.28)	0.14 (-0.05, 0.33)
	Model 3	-0.04 (-0.27, 0.18)	0.11 (-0.03, 0.25)	0.02 (-0.25, 0.28)	0.14 (-0.05, 0.33)
	Outcome	Pure Tone Audiometry *			
Sniffin' Sticks (1 unit worse) †	Model 1	0.34 (-0.18, 0.86)	0.38 (-0.28, 1.03)	0.87 (-0.41, 2.14)	0.47 (-0.34, 1.29)
	Model 2	0.33 (-0.19, 0.85)	0.37 (-0.28, 1.04)	0.73 (-0.58, 2.04)	0.66 (-0.13, 1.45)
	Model 3	0.32 (-0.21, 0.85)	0.33 (-0.34, 1.00)	0.85 (-0.51, 2.21)	0.60 (-0.20, 1.40)

Note. All vision measures are in the scale of 0.1 unit. For presenting and corrected distance visual acuity, and near visual acuity, 0.1 logMAR represented 5 letters or 1 line on the ETDRS chart and, 1 line on the MNRead chart, respectively. For contrast sensitivity, 0.1 log represented 2.5 letters on the MARS chart.

Model 1: adjusted for age.

Model 2: adjusted for age, education level.

Model 3: adjusted for age, education level, ever smoker status, diabetes, and hypertension.

* Greater impairment is represented by higher values.

† Scale has been reversed such that greater impairment is represented by higher values.

‡ indicates significant exposure : community/race : sex interaction term in the relevant model, using the full cohort, additionally adjusted for sex, community/race, exposure : sex interaction, exposure : community/race interaction, community/race : sex interaction, and exposure : community/race : sex interaction.

logMAR, log_{10}[minimum angle of resolution]; log, logarithm

§ $p < 0.05$

Supplementary Table S3. Linear regression estimates (95% confidence interval) of the associations of different sensory functions, stratified by community/race-sex, which are statistically significant in at least one community/race-sex stratum, after performing multiple imputation by chained equations (MICE)

	Community/ race	Jackson/Black women	Washington County/White women	Jackson/Black men	Washington County/White men
n		354	314	141	247
Exposure	Outcome	Quick Speech-in-Noise †			
Near visual acuity (0.1 logMAR worse) *	Model 1	0.22 (0.002, 0.44) §	0.48 (0.16, 0.79) §	0.27 (0.09, 0.45) §	0.12 (-0.22, 0.46)
	Model 2	0.15 (-0.002, 0.30)	0.38 (0.07, 0.69) §	0.21 (0.03, 0.40) §	-0.06 (-0.39, 0.28)
	Model 3	0.16 (0.02, 0.29) §	0.39 (0.07, 0.70) §	0.22 (0.04, 0.41) §	-0.05 (-0.39, 0.28)
Contrast sensitivity (0.1 log worse) †	Model 1	0.40 (0.06, 0.74) §	0.53 (0.23, 0.82) §	0.09 (-0.36, 0.54)	0.28 (-0.22, 0.78)
	Model 2	0.31 (-0.007, 0.63)	0.46 (0.17, 0.75) §	-0.07 (-0.53, 0.39)	0.19 (-0.29, 0.67)
	Model 3	0.32 (0.02, 0.63) §	0.47 (0.18, 0.75) §	-0.07 (-0.57, 0.42)	0.19 (-0.29, 0.67)
	Outcome	Sniffin' Sticks †			
Presenting distance visual acuity (0.1 logMAR worse) *	Model 1	0.14 (-0.47, 0.74)	0.19 (0.007, 0.37) §	0.18 (-0.26, 0.61)	0.18 (0.02, 0.33) §
	Model 2	0.11 (-0.49, 0.71)	0.19 (0.005, 0.37) §	0.13 (-0.33, 0.59)	0.19 (0.03, 0.34) §
	Model 3	0.10 (-0.50, 0.71)	0.17 (-0.007, 0.35)	0.08 (-0.42, 0.58)	0.17 (0.009, 0.33) §
Near visual acuity (0.1 logMAR worse) *	Model 1	0.11 (-0.11, 0.34)	0.14 (-0.01, 0.29)	0.12 (0.05, 0.19) §	0.16 (0.03, 0.28) §
	Model 2	0.10 (-0.10, 0.30)	0.13 (-0.02, 0.29)	0.09 (0.02, 0.17) §	0.19 (0.06, 0.31) §
	Model 3	0.10 (-0.10, 0.30)	0.12 (-0.03, 0.27)	0.09 (0.01, 0.16) §	0.18 (0.06, 0.31) §
	Outcome	Quick Speech-in-Noise †			
Sniffin' Sticks (1 unit worse) †	Model 1	0.32 (0.14, 0.50) §	0.13 (-0.12, 0.38)	0.71 (0.05, 1.37) §	-0.09 (-0.46, 0.28)
	Model 2	0.24 (0.07, 0.40) §	0.11 (-0.12, 0.34)	0.62 (-0.02, 1.26)	-0.002 (-0.36, 0.36)
	Model 3	0.24 (0.08, 0.40) §	0.13 (-0.11, 0.37)	0.67 (0.04, 1.31) §	0.02 (-0.34, 0.39)

Note. All vision measures are in the scale of 0.1 unit. For presenting distance and near visual acuity, 0.1 logMAR represented 5 letters or 1 line on the ETDRS chart, and 1 line on the MNRead chart, respectively. For contrast sensitivity, 0.1 log represented 2.5 letters on the MARS chart.

Model 1: adjusted for age.
Model 2: adjusted for age, education level.
Model 3: adjusted for age, education level, ever smoker status, diabetes, and hypertension.
* Greater impairment is represented by higher values.
† Scale has been reversed such that greater impairment is represented by higher values.
logMAR: $\log_{10}$[minimum angle of resolution]; log: logarithm
§ $p < 0.05$

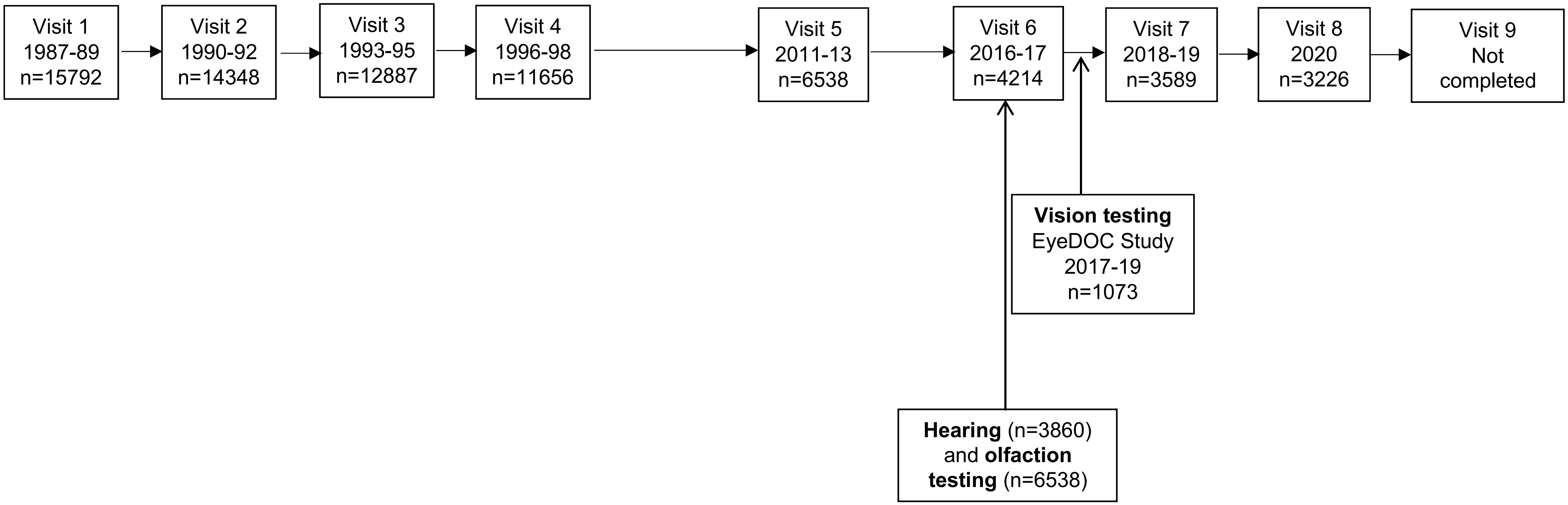

Supplementary Figure S1. Flowchart of the Atherosclerosis Risk in Communities Study and the ancillary studies used in our study**.**

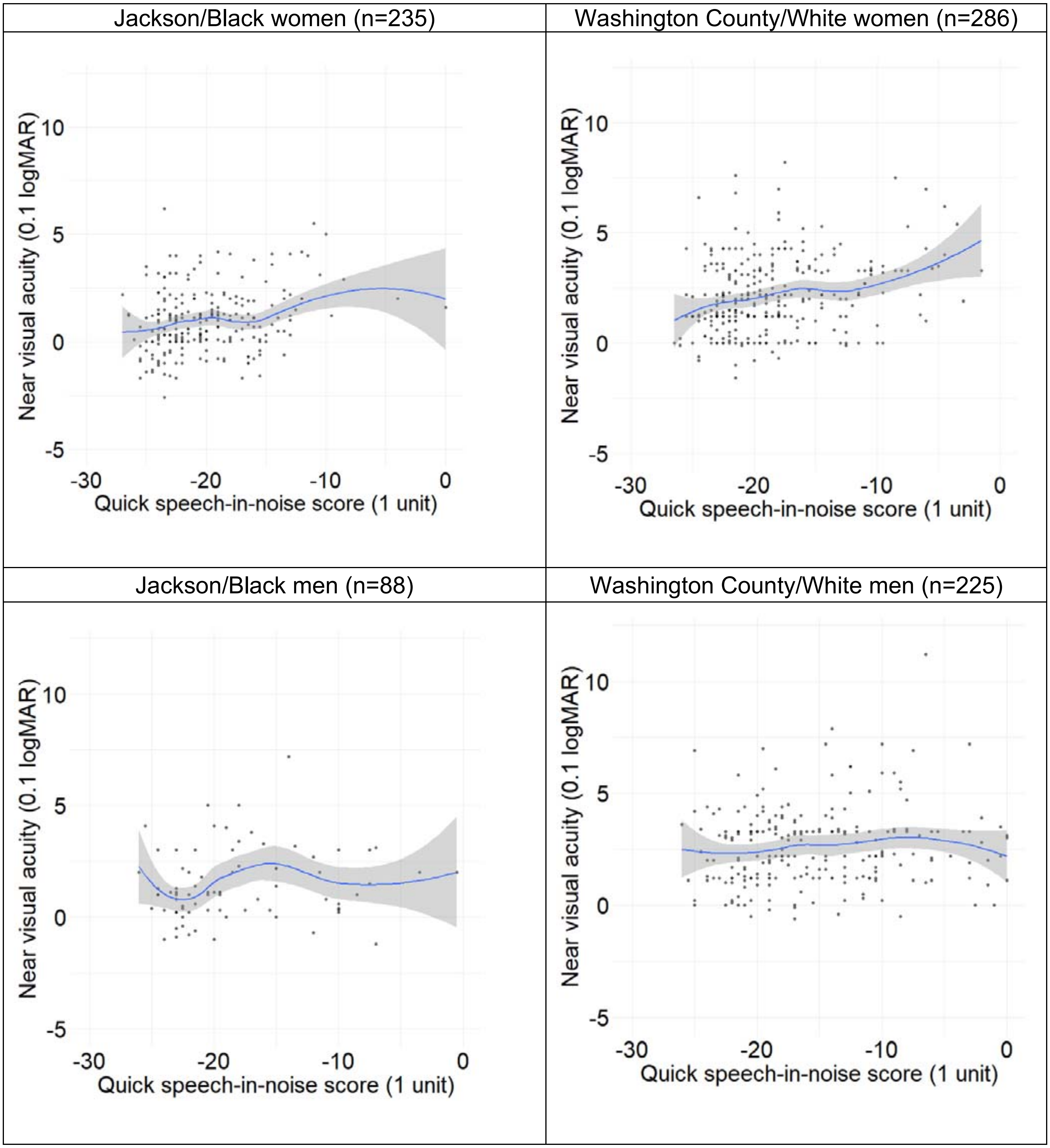

Supplementary Figure S2. Scatter plots with Lowess smoothing (95% confidence interval) for near-visual acuity with central auditory processing (Quick Speech-in-Noise), stratified by community/race-sex**.**

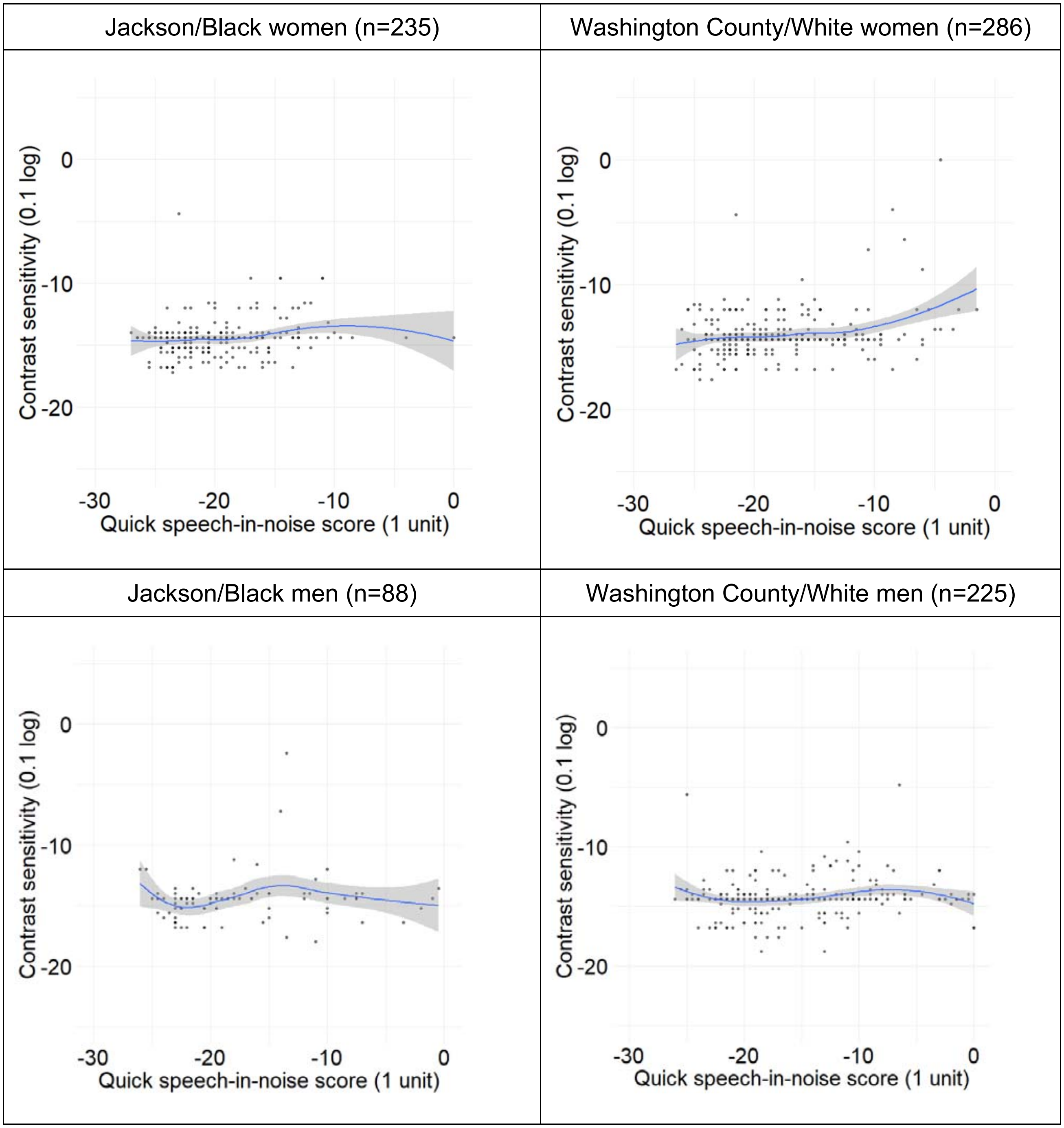

Supplementary Figure S3. Scatter plots with Lowess smoothing (95% confidence interval) for contrast sensitivity with central auditory processing (Quick Speech-in-Noise), stratified by community/race-sex**.**

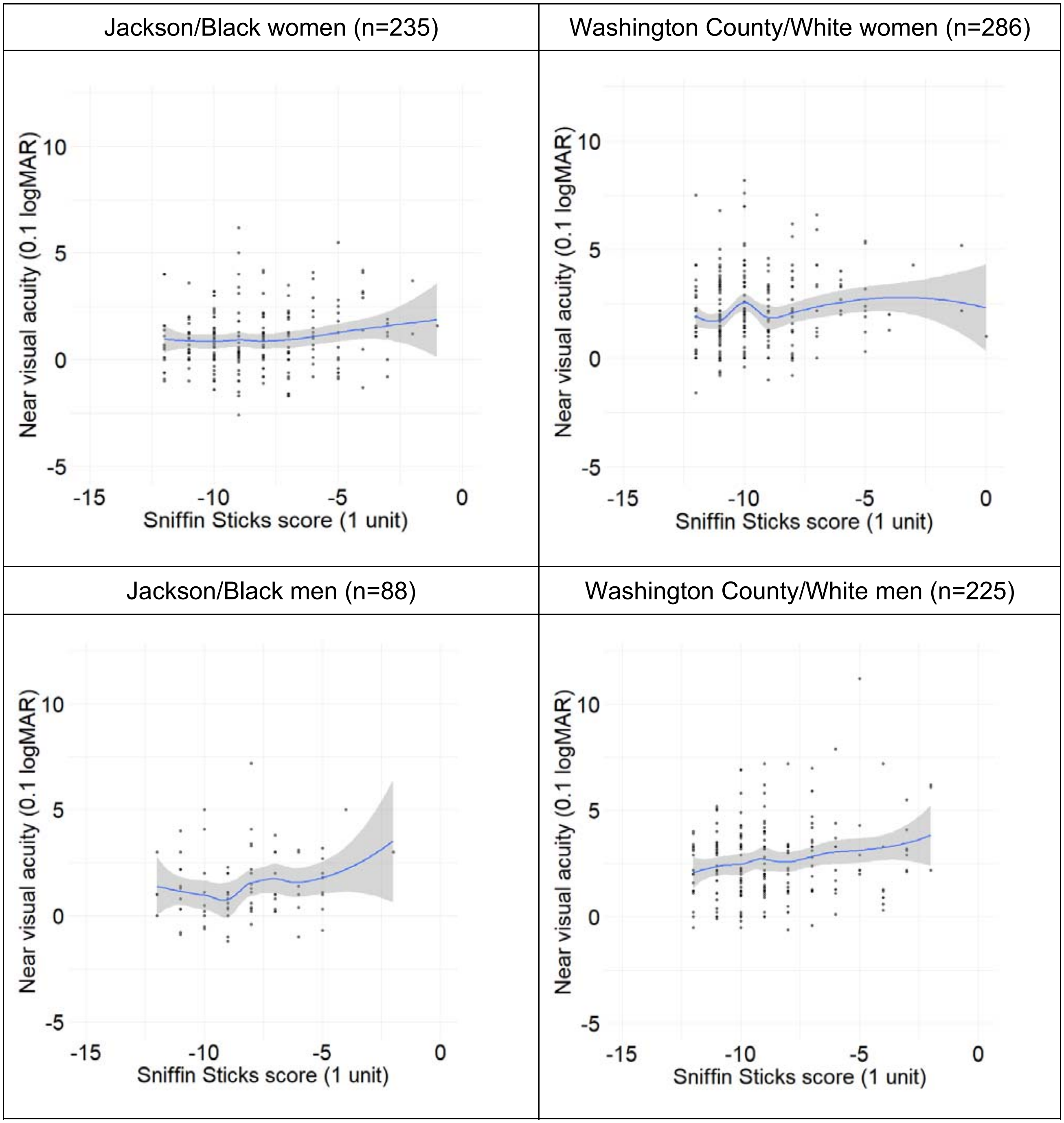

Supplementary Figure S4. Scatter plots with Lowess smoothing (95% confidence interval) for near-visual acuity with olfaction (Sniffin' Sticks), stratified by community/race-sex**.**

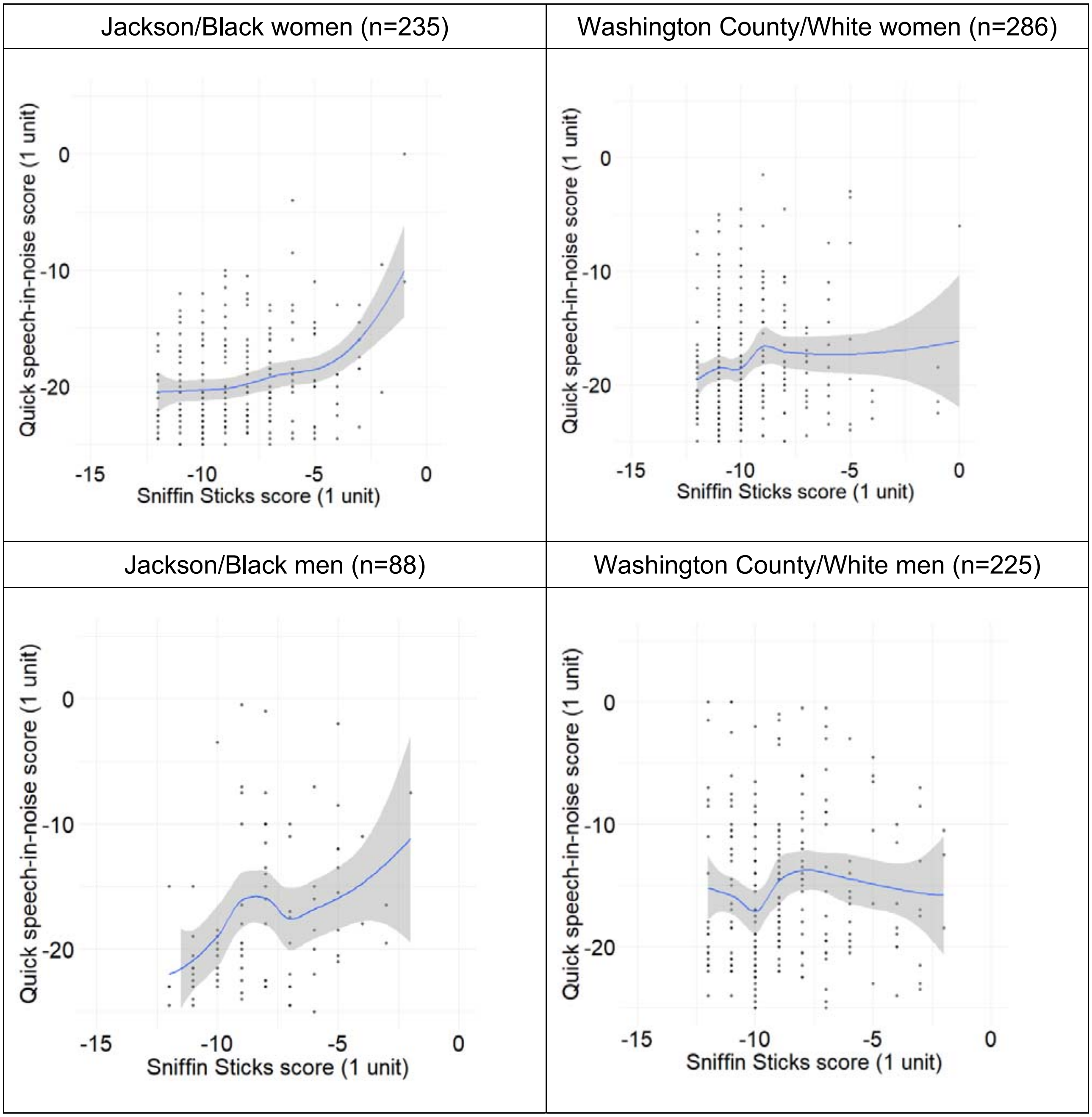

Supplementary Figure S5. Scatter plots with Lowess smoothing (95% confidence interval) for olfaction (Sniffin' Sticks) with central auditory processing (Quick Speech-in-Noise), stratified by community/race-sex**.**
